# Interaction between drinker density and cow social dominance affects drinking behavior

**DOI:** 10.3168/jdsc.2023-0479

**Published:** 2023-12-09

**Authors:** E. Nizzi, C. Hurtaud, A. Boudon

**Affiliations:** PEGASE, INRAE, Institut Agro, 35590, Saint Gilles, France

## Abstract

Dairy cow welfare and milk production levels can be negatively affected when animals lack access to sufficient amounts of good-quality drinking water. Farms vary greatly in how well they meet this standard. Here, we explored how the interaction between drinker density and social dominance level influenced the drinking behaviors of a group of 40 mid-lactation cows. We recorded cow drinking behavior and competitive outcomes (i.e., number of replacements) at the drinkers during the final 5 d of 2 drinker density treatments, applied successively (12 drinkers, then 4 drinkers available). Four social dominance categories were defined by applying the quartile classification method to the normalized David's score obtained from the low-density treatment data (NormDS values). The high- and low-density treatments used approximately 56 (±16) versus 119 (±31) replacements per drinker per day. Subordinate cows were lighter and younger than dominant cows. Dominant cows drank around 5 L more per day in the low- versus high-density treatment. Furthermore, both drinking rate and daily drinking frequency were higher in the low-density treatment, with the most pronounced differences seen for subordinate cows. In the low-density treatment, mid-subordinate cows shifted their drinking times, visiting the drinkers 1 to 2 h after peak water consumption by the group. The results for the low-density treatment thus indicate that the cows were experiencing more intense competition for water, leading to behavioral responses that were related to social dominance. Our findings underscore the need to more broadly explore the effects of increasing drinker numbers under a variety of rearing conditions to provide farmers with better technical recommendations.

## Highlights

• Low-density treatment generated competition for water.
• Low-density treatment increased replacements, drinking rate, and drinking frequency.
• Dominant cows monopolized drinkers and drank 5 L more in the low-density treatment.
• Subordinate cows showed greater variations in drinking rate and frequency.
• Mid-subordinate cows shifted their drinking times to avoid peak watering periods.

## Graphical Abstract

**Figure:**
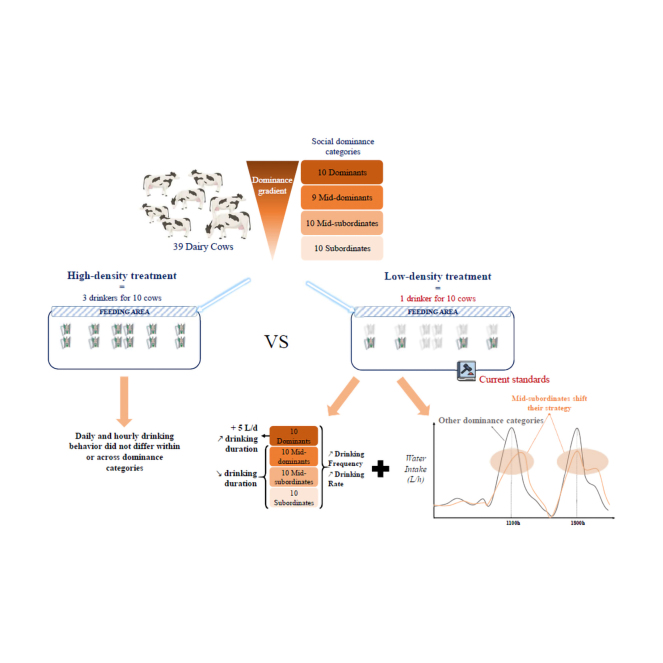
**Summary** Access to sufficient drinking water is an essential part of animal welfare. It is known that rearing conditions and social dominance hierarchies can influence cow drinking behavior. Here, we explored the interaction between drinker numbers and social dominance for a group of 40 cows. When cows were given the number of drinkers specified by current official standards (1 drinker per 10 cows), dominant cows monopolized access and drank 5 L more than when drinker density was higher (1 per 3.3 cows). Furthermore, at the lower drinker density, drinking frequency and rate increased for cows of all dominance levels but particularly for subordinates; mid-subordinates shifted to off-peak drinking times. Our results illustrate that current standards for drinker numbers can lead to competition among animals, whose behavioral responses may depend on their level of social dominance. In conclusion, technical guidelines should be carefully revisited with a view to ensuring animal welfare on dairy farms.

Access to water of sufficient quantity and quality is essential to the welfare of dairy cows (Welfare Quality, 2009). A survey evaluating more than 130 French dairy farms following the Welfare Quality protocol (2009) found that farms varied greatly in their degree of compliance and that a considerable proportion of farms failed to provide adequately for their cows' daily watering needs (de Boyer des Roches et al., 2012). Such differences are growing more dramatic in the context of climate change and the increasing frequency of heat waves.

Official standards for watering animals on dairy farms exist (NRC, 2001; Welfare Quality, 2009). However, they specify a minimum number of drinkers, minimum water flow rate, or optimal drinker location, but they say nothing about means of ensuring equal access to water for all cows. Instead, the standards simply require that 10% of a herd's cows be able to drink at the same time. The basis for these standards was first articulated in research by Castle and Thomas (1975), which characterized the drinking behavior of 14 dairy herds (n = 840 lactating cows) in England and Wales over a 6-mo period. The NRC (2001) and Welfare Quality (2009) standards are a simplified, less stringent version of the initial recommendations made by Castle and Thomas (1975).

Little research has examined the effects of drinker numbers on watering behavior within dairy cow herds. One study (Andersson, 1987) has compared the behavior of 2 small groups (~15 cows) with access to 1 or 2 drinking bowls. When 2 bowls were available, the animals were found either close together or far away from each other. Furthermore, although drinker visits were more frequent when 2 bowls were present, bowl number did not influence total water intake. Given the technology available at the time, it was impossible to interpret individual drinking behavior at a fine scale

Access to water is essential to animal well-being, whether at the group or at the individual level. However, an important distinction must be made, namely that “the concept of well-being applies to the mental dimension of the animal's experience of its environment” (ANSES, 2018, p. 16). In their review, Jensen and Vestergaard (2021) highlighted the importance of considering cow dominance hierarchies and the influence of cow numbers when characterizing the effects of drinker density, especially when developing standards to ensure that all animals of the herd have unrestricted access to water. Although research has yet to consider the interaction between drinker numbers and individual behavior within dairy cow herds, past studies have highlighted the importance of animal social rank in behavioral responses to reduced feeder availability (Olofsson, 1999). At present, highly precise tools exist that can better measure individual variability in drinking behavior. Recent work has validated the utility of a connected drinker that can collect data on competitive outcomes between cows and social dominance (Foris et al., 2019).

The objective of this study was to explore how the interaction between drinker numbers and social dominance could affect watering behavior in a group of 40 cows housed in a freestall barn using connected drinkers. We hypothesized that reducing drinker numbers would increase competitive interactions among animals, leading to more replacements at drinkers. In this vein, we predicted that lower drinker numbers would also amplify behavioral differences based on cow social ranking, such that dominant cows might even limit drinker visits by subordinate cows.

Our experiment was carried out at INRAE's Experimental Facility for Dairy Nutrition and Physiology (IEPL, Le Rheu, France; https://doi.org/10.15454/yk9q-pf68, protocol L2302) over 3 wk in February 2023 using a group of 40 mid-lactation Prim'Holstein cows (16 primiparous and 24 multiparous). During the first week (February 1–8), cows had access to 12 drinkers (high-density treatment). During the second and third weeks (February 9–22), cows had access to 4 drinkers (low-density treatment). In the low-density treatment, drinker numbers followed current standards (1 drinker per 10 cows); in the high-density treatment, 3 times as many drinkers were available (3 drinkers per 10 cows).

At the start of the experiment, mean parity (±SE) in the group was 2.1 (±1.2) and mean (±SE) milk yield (**MY**) in kg/d was 31.8 (±5.1). Animals were reared following animal care guidelines issued by the French Ministry of Agriculture and Sovereignty and in accordance with French legislation (Decree 2001-464, May 29, 2001; agreement number E 35-275-23; project no. APAFIS 39513-2022112207567178 v3). Cows were housed in a freestall pen containing 40 individual lying stalls. Each cow was allocated a specific feeder, where access was controlled by a radio-frequency identification system. Twice a day (at 0800 and 1600 h), an automated feed system deposited a TMR composed of 65% corn silage, 12.5% soybean meal, 10% dehydrated alfalfa, and 12.5% concentrate (% DM) as well as 200 g of mixed minerals and vitamins. Cows were fed ad libitum (10% refusal) and milked twice a day (at 0730 and 1530 h). After milking, they remained blocked at the feed fence for around 1.5 h. There were no drinkers in the milking parlor or along the path from the milking parlor to the pen.

The interconnected drinkers (La Buvette, Charleville-Mézières, France) were installed in our experimental facilities in 2018, which meant that the study cows were already familiar with the watering system. The drinkers are 3-L bowls constructed of high-density polyethylene. Both level and flow are automatically maintained via a float at the rear of the drinker. Only one cow can water at a time because drinker access is restricted by the presence of 2 rigid sides. The radio-frequency identification system on the left of the drinker recorded the cow's transponder number. Using pulse measurement and automated software (pulse = 0.0248 L), a flow meter used the water flow (±12 L/min) to calculate cow water intake. Also recorded for each visit were the time of day and drinking duration. The drinking data were centralized using a Blue Intelligence system (La Buvette, Charleville-Mézières, France) and exported as Microsoft Excel files.

Eight cameras distributed across the pen continuously recorded the dairy cows' daily interactions at the drinkers between 0530 h and 1030 h over the entire experiment. The cows were marked with symbols on their backs and sides to facilitate their identification during video analysis, which was performed using VS Player software (2019 Hangzhou Hikvision Digital Technology Co. Ltd.). The first 3 d of each treatment were considered to be an adaptation period. Replacements at the drinkers were then examined for the next 5 d. A replacement was defined as occurring when, within a 60-s interval, one cow directed aggressive behavior toward a second cow, resulting in the latter's departure of the drinker.

All the statistical analyses were performed using R (v. 4.2.1; R Core Team, 2022). We established dominance hierarchies for the cows using 5-d sets of replacement data (high-density: February 4–8; low-density: February 12–16). We applied the David's score (**DS**) method (de Vries et al., 2006) to the resulting dominance matrix to calculate each cow's normalized dominance score (**NormDS**). The cows were ranked in decreasing order based on their NormDS (i.e., higher NormDS = more socially dominant, vs. lower NormDS = less socially dominant). One cow was removed because of a slip in the alley, and her data were excluded from the analysis.

The ranks obtained in the low-density treatment—that is, the most competitive—were used to define cow dominance categories. These ranks were employed to divide the cows up into 4 dominance categories using a quartile classification method (Q1 = 25th percentile, Q2 = 50th percentile, Q3 = 75th percentile). Considering the lack of knowledge on drinker number studies, statistic power was not been based on previous studies. We considered that a 40-cow sample size in this pilot study was enough to show the differences in water intake between dominance categories, considering an effect size of 1.1, with a power of 0.95 and a type I error of 0.05 (estimated sample size = 38 cows; GPower, Universität Kiel, Germany).

Data were analyzed per cow. For each analysis, we used the 4 last days of each treatment period (i.e., high-density treatment: February 5–8; low-density treatment: February 19–22). We examined how dominance category and treatment affected individual water intake, drinking duration, drinking frequency, drinking rate, DMI, MY, ratio of water intake to DMI, and ratio of water intake to MY. We examined the influence of dominance category (fixed effect; df = 3) on parity and BW using a linear model (lm model). We used a linear model (lmer model; Kuznetsova et al., 2017) in which dominance category (df = 3), treatment (df = 1), and their interaction (df = 3) were fixed effects; day (df = 3) and cow identification number (df = 38) were random effects. The effect of the treatment on hourly water intake was analyzed using a linear model in which the hour of the day (df = 23), dominance category (df = 3), and their interaction were fixed effects (df = 69); day (df = 3) and cow identification number (df = 38) were random effects. Given the exploratory nature of our study, the α level was not corrected for multiple comparisons. We visually examined residue normality via Q-Q plot and tested variance homogeneity across the dominance categories via Levene test (Fox and Weisberg, 2019). We reported results as significant when *P* ≤ 0.05 and as marginally insignificant when 0.05 < *P* ≤ 0.10.

Herein, we report mean values (±SD). Mean temperatures in the pen were 9.2°C (±3.4) and 11.3°C (±2.7) over the last 4 d of the high-density and low-density treatments, respectively. The mean DM content of the TMR was 57.3% (±0.7) in the high-density treatment and 56.3% (±0.5) in the low-density treatment. During the high-density treatment, 2 primiparous cows were observed to have digital dermatitis (requiring no antibiotics); no health concerns were seen during the low-density treatment. Mean DIM was 139 d (±6) in the high-density treatment and 152 d (±6) in the low-density treatment. There were 281 replacements during the high-density treatment and 592 replacements during the low-density treatment.

There were 10 cows in the dominant group, 9 cows in the mid-dominant group, 10 cows in the mid-subordinate group, and 10 cows in the subordinate group; within these groups, 2, 3, 4, and 7 cows were primiparous, respectively. Parity and BW were significantly different for the subordinate versus the dominant cows (parity: 1.4 ± 0.4 vs. 2.9 ± 0.4, *P* = 0.022; BW: 605 kg ± 21 vs. 705 kg ± 21, *P* = 0.015; Table 1). No effect of dominance category was detected on DMI. In the low-density treatment, MY was significantly greater for dominant cows (32.4 kg ± 1.4) than for subordinate cows (27.9 kg ± 1.4; *P* = 0.008). This MY of dominant cows (32.4 kg ± 1.4) was also significantly higher than for the mid-subordinate and subordinate cows (29.2 kg ± 1.4 and 28.4 kg ± 1.4, respectively; *P* = 0.008) of the high-density treatment.

**Table 1:** Influence of treatment and dominance category on dairy cow physiology, feeding behavior, and drinking behavior (LSM ± SE)^1^

Variable	High-density treatment				Low-density treatment				*P*-value		
	DOM (n = 10)	MID-DOM (n = 9)	MID-SUB (n = 10)	SUB (n = 10)	DOM (n = 10)	MID-DOM (n = 9)	MID-SUB (n = 10)	SUB (n = 10)	DomC	Treat	DomC × Treat
Parity^2^	2.9^a^ ± 0.4	2.3^ab^ ± 0.4	1.6^ab^ ± 0.4	1.4^b^ ± 0.4					0.022	ND	ND
BW^2^ (kg)	705^a^ ± 21	677^ab^ ± 22	660^ab^ ± 21	605^b^ ± 21					0.015	ND	ND
MY (kg)	31.7^AB^ ± 1.4	29.3^AB^ ± 1.5	29.2^B^ ± 1.4	28.4^B^ ± 1.4	32.4^A^ ± 1.4	29.5^AB^ ± 1.5	28.5^AB^ ± 1.4	27.9^B^ ± 1.4	0.388	0.152	0.008
DMI (kg)	24.6 ± 0.9	24.1 ± 0.9	23.4 ± 0.9	22.3 ± 0.9	25.3 ± 0.9	24.2 ± 0.9	23.3 ± 0.9	22.5 ± 0.9	0.248	0.074	0.206
Water intake (L)	79.5^B^ ± 4.2	82.1^AB^ ± 4.8	82.2^AB^ ± 4.2	77.2^BC^ ± 4.2	84.2^A^ ± 4.2	82.3^AB^ ± 4.8	82.6^AB^ ± 4.2	75.2^C^ ± 4.2	0.819	0.005	0.041
Water intake/MY (L/kg)	2.52^b^ ± 0.1	2.81 ± 0.1	2.83^a^ ± 0.1	2.73 ± 0.1	2.60^b^ ± 0.1	2.78 ± 0.1	2.92^a^ ± 0.1	2.72 ± 0.1	0.029	0.218	0.413
Water intake/DMI (L/kg)	3.23 ± 0.1	3.40 ± 0.1	3.50 ± 0.1	3.46 ± 0.1	3.33 ± 0.1	3.36 ± 0.1	3.55 ± 0.1	3.34 ± 0.1	0.231	0.238	0.164
Drinking duration (min)	9.30 ± 0.6	9.49 ± 0.7	9.90 ± 0.6	9.02 ± 0.6	9.67 ± 0.6	9.39 ± 0.7	9.81 ± 0.6	8.35 ± 0.6	0.786	0.162	0.051
Drinking frequency (no. of visits)	10.2 ± 1.3	10.1 ± 1.3	9.60 ± 1.3	9.62 ± 1.3	11.5 ± 1.3	12.6 ± 1.3	10.9 ± 1.3	12.5 ± 1.3	0.979	0.075	0.274
Drinking rate (L/min)	8.67 ± 0.3	8.63 ± 0.3	8.51 ± 0.3	8.62 ± 0.3	8.86 ± 0.3	8.82 ± 0.3	8.67 ± 0.3	9.07 ± 0.3	0.976	0.057	0.116

a,b Different lowercase superscripts indicate the presence of significant differences (*P* < 0.05) after post hoc comparisons of dominance categories.

A–C Different uppercase superscripts indicate the presence of significant differences (*P* < 0.05) after the post hoc comparisons of different dominance categories by treatment.

1 DOM = dominant cows; MID-DOM = mid-dominant cows; MID-SUB = mid-subordinate cows; SUB = subordinate cows; DomC = dominance category; Treat = treatment; MY = milk yield.

2 Parity and BW reported here were measured once for each animal at the beginning of the experiment. The sole predictor variable in the mixed model was dominance category.

Water intake was higher in the low-density treatment than in the high-density treatment (*P* = 0.005), a pattern seemingly driven by the dominant cows (84.2 L ± 4.2 vs. 79.5 L ± 4.2, respectively; treatment-by-dominance interaction: *P* = 0.041). Furthermore, subordinate cows consumed less water than dominant cows in the low-density treatment (72.5 L ± 4.2 vs. 84.2 L ± 4.2; *P* = 0.041); such was not the case in the high-density treatment. The ratio of water intake to MY was lower for the dominant cows than for the mid-subordinate cows for both treatments (means of 2.56 L/kg ± 0.1 and 2.88 L/kg ± 0.1, respectively; *P* = 0.029). Effects of treatment or dominance category were absent for the ratio of water intake to DMI. Drinking duration tended to be shorter for subordinate cows in the low-density treatment than for cows in all the other combinations of groups (treatment-by-dominance interaction: *P* < 0.051). Drinking frequency tended to be greater in the low-density treatment (11.9 ± 1.3) than in the high-density treatment (9.9 ± 1.3; *P* = 0.075). The same trend was seen for drinking rate (low-density = 8.9 L/min ± 0.03, high-density = 8.6 L/min ± 0.3; *P* = 0.057).

In both treatments, water consumption showed prominent peaks at 1100 h and 1800 h (Figure 1; hour of day: *P* < 0.001). Social dominance had no effect on water intake in either treatment. No interaction was detectable between dominance category and hour of day (*P* = 0.645) in the high-density treatment, but there was in the low-density treatment (*P* = 0.007). In the low-density treatment at 1100 h, mid-subordinate cows drank significantly less water than cows in all the other dominance categories (mean: 10.1 L ± 1.1 vs. 17.7 L ± 1.1, respectively; *P* < 0.001). At 1200 h, subordinate cows drank significantly less than dominant and mid-subordinate cows (mean: 6.1 L ± 1.1 vs. 11.2 L ± 1.1, respectively; *P* < 0.05). At 1800 h, mid-subordinate cows consumed 5.3 L less than dominant cows (12.6 L ± 1.1 vs. 17.9 L ± 1.1, respectively; *P* < 0.01). Two hours later, subordinate cows consumed approximately half as much water as mid-subordinate cows (4.7 L ± 1.1 vs. 8.8 L ± 1.1, respectively; *P* < 0.05).

**Figure 1:**
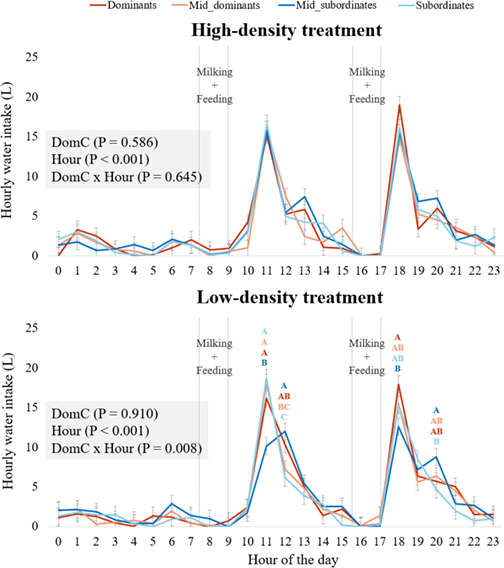
Hourly water intake by the cows over the course of a day for both treatments and each dominance category. The x-axis represents the hours of the day (e.g., 10 = water intake by cows between 1000 and 1059 h). Data were averaged by social category and hour and related to the number of cows. Analysis of variance was performed for each treatment period. DomC = dominance category; Hour = hour of the day. Letters A to C indicate differences (*P* < 0.05) after the post hoc comparisons between categories within hour, and error bars represent SE.

Although a confounding influence of time on the effect of our treatments is possible, given that treatments took place successively, our results nonetheless strongly suggest that changes in cow drinking behavior were mainly due to drinker number. Indeed, in dairy cows, the main variable associated with water intake is MY, which is partially correlated with DMI diet composition (namely DM content and CP content), and ambient temperature (INRA, 2018). However, we designed our experiment so as to limit the variability of such factors and thus maximize the treatment effects. Minimal differences in lactation stage occurred across the 3 wk of the experiment, not dramatic enough to affect MY or DMI in mid-lactating cows (INRA, 2018). Two variables might have been difficult to control, but fortunately remained consistent across treatments: diet composition and DM content. Furthermore, pen temperature differed by just 2°C between treatments, which was unlikely to affect water intake given the cows' overall thermal conditions (INRA, 2018). The cows had been exposed to their housing conditions for several months before our study. The experimental group was put together 5 d before our research began, allowing sufficient time for a social dominance hierarchy to become established (Kondo and Hurnik, 1990).

Compared with Foris et al. (2019), we observed a large number of replacements at the drinkers. This result can largely be explained by the fact that, in our study, the cows spent more than an hour at the feed fence after the milking process. Such is a common practice used to limit the risk of mastitis because it prevents the cows from lying down too soon after milking, which can cause teat contamination (Peeler et al., 2000). This practice and the cows' concomitant access to the feeding troughs, limited competition at the feed fence. Furthermore, the cows were strongly primed to experience “postprandial thirst” (Langhans et al., 1995) when the feed fence was opened.

During the low-density treatment, the number of replacements was high—more than 20 interactions per individual—which is considered optimal for establishing a reliable social dominance hierarchy (Sánchez-Tójar et al., 2018). In previous studies on social competition (Huzzey et al., 2014) or social dominance (Foris et al., 2019), replacements were measured at feeders, where intense competition generates abundant interactions per cow within a few days' time. Under our specific rearing conditions, most competitive interactions occurred at the drinkers.

The large percentage of primiparous cows among the subordinate cows (70%) likely explains the trend toward lower DMI in this dominance category. In contrast, only 20% of the dominant cows were primiparous. This finding is consistent with the idea that parity influences social ranking in the herd (Val-Laillet et al., 2009). The subordinate cows also weighed significantly less than the dominant cows, which makes sense given that height and BW shape social ranking as well (Phillips and Rind, 2001). Thus, differences in MY between dominant and subordinate cows can be explained, at least in part, by the physiologically related increase in MY from the first to the second lactation period, attributable to the increases in DMI and BW that occur over the course of development (Knight and Wilde, 1993). In this study, each cow had its own specific feeder. Thus, we can safely assume that competition for feed was unlikely to drive differences in DMI or MY between cow parities.

Here, the number of replacements more than doubled between the first and second treatment, clearly illustrating that competition for access to water had increased. The latter was also reflected in other variables related to drinking behavior. Drinking frequency and rate also tended to increase across the board during the low-density treatment, with the largest differences seen in the subordinate cows. These results can be explained by the increase in replacements, a situation in which replaced animals had to return to the drinkers numerous times and drink quickly to meet their need for water. This increasing competition could indicate lower animal welfare standards because cows need to enter in the competition and received negative interactions to access water. Such shifts in behavior were also seen in a study in which different levels of feeding competition were created using 3 treatments: overstocking, temporal feed restriction, and both simultaneously. The result was a high number of displacements at the feeder, with cows eating more quickly and returning to the feeder more frequently (Collings et al., 2011).

Finally, in the low-density treatment, we expected that the subordinate cows would have less access to the drinkers and thus lower water intake levels than in the high-density treatment. However, contrary to our predictions, the subordinate cows drank the same amount of water during both treatments, and the dominant cows drank 5 L more per day in the low-density treatment. This result was consistent with the findings of Coimbra et al. (2012), who observed that certain animals competitively monopolized water resources. Although the subordinate cows in this study might have succeeded in drinking enough water, their changed drinking behavior in the low-density treatment is reflective of the greater level of competition.

During both treatments, we saw pronounced peaks in water consumption in the morning and afternoon, corresponding to the opening of the feed fences, one hour after milking and the arrival of the cows' fresh feed. The peaks in our study were much more marked than those in Cardot et al. (2008), where cows could drink when they wanted after milking and feed delivery. As a result, the cows probably spent substantial time competing for feed, desynchronizing their visits to the drinker.

In the low-density treatment, the mid-subordinate cows seemed to drink at different times than the cows in the other dominance categories (i.e., 1–2 h after peak water consumption by the group). Past research has shown that, during periods of intense competition, certain individuals shift their watering times to avoid negative interactions with other individuals (McDonald et al., 2019). We suggest that some mid-subordinate cows adopted this strategy during the most competitive moments of the low-density treatment. A recent critical review of the methods used to determine social dominance (Krahn et al., 2023) highlighted that, during resource competition, the number of agonistic interactions can depend on an animal's motivation to access the resource (Coimbra et al., 2012). We think the subordinate and mid-subordinate cows developed different strategies during moments of intense competition: the subordinates were more motivated to access the drinkers, facing a greater likelihood of replacements and the need for more frequent visits, whereas the mid-subordinate cows shifted their drinking times. This drinking shifting by mid-subordinates during the low-density treatment may indicate an inability to satisfy their motivated drinking behavior, and so a poorer animal welfare outcome. We recommend that future research explore the relationship between social dominance and more detailed facets of drinking behavior such as levels of motivation, signs of frustration, or presence of compensatory drinking.

In conclusion, we found that drinker availability influenced the interaction between social dominance and cow drinking behavior. Reducing the number of drinkers increased the number of replacements, water intake by the dominant cows, and drinking frequency and rate of the subordinate cows. It also resulted in a change in the drinking dynamics of mid-subordinate cows, who shifted to drinking water 1 to 2 h after peak consumption by the group. We wish to underscore that the low-density treatment corresponded to current watering standards for commercial farms. Yet, competition for water was far from negligible. Indeed, we observed dramatic differences in drinking behavior between treatments, especially for dominant versus subordinate cows. This finding highlights that researchers must better characterize the effect of social dominance hierarchies on access to drinkers to improve technical watering requirements. The current welfare standard of 1 drinker per 10 cows, based on researches when cows produced much less milk, may not be appropriate for today's dairy farms from an animal welfare point of view.
